# The Inflammasome Adaptor ASC Intrinsically Limits CD4^+^ T-Cell Proliferation to Help Maintain Intestinal Homeostasis

**DOI:** 10.3389/fimmu.2019.01566

**Published:** 2019-07-15

**Authors:** Hanif Javanmard Khameneh, Keith Weng Kit Leong, Andrea Mencarelli, Maurizio Vacca, Bezaleel Mambwe, Kurt Neo, Alice Tay, Francesca Zolezzi, Bernett Lee, Alessandra Mortellaro

**Affiliations:** ^1^Singapore Immunology Network (SIgN), Agency for Science, Technology and Research (A^*^STAR), Singapore, Singapore; ^2^Department of Infection, Immunity and Cardiovascular Diseases, The University of Sheffield, Sheffield, United Kingdom; ^3^San Raffaele Telethon Institute for Gene Therapy (SR-Tiget), IRCCS San Raffaele Scientific Institute, Milan, Italy

**Keywords:** ASC, inflammasome, CD4^+^ T cells, colitis, inflammation

## Abstract

The inflammasome is a multi-protein complex that mediates proteolytic cleavage and release of the pro-inflammatory cytokines IL-1β and IL-18, and pyroptosis—a form of cell death induced by various pathogenic bacteria. Apoptosis-associated speck-like protein containing a CARD (ASC) has a pivotal role in inflammasome assembly and activation. While ASC function has been primarily implicated in innate immune cells, its contribution to lymphocyte biology is unclear. Here we report that ASC is constitutively expressed in naïve CD4^+^ T cells together with the inflammasome sensor NLRP3 and caspase-1. When adoptively transferred in immunocompromised Rag1^−/−^ mice, Asc^−/−^ CD4^+^ T cells exacerbate T-cell-mediated autoimmune colitis. Asc^−/−^ CD4^+^ T cells exhibit a higher proliferative capacity *in vitro* than wild-type CD4^+^ T cells. The increased expansion of Asc^−/−^ CD4^+^ T cells *in vivo* correlated with robust TCR-mediated activation, inflammatory activity, and higher metabolic profile toward a highly glycolytic phenotype. These findings identify ASC as a crucial intrinsic regulator of CD4^+^ T-cell expansion that serves to maintain intestinal homeostasis.

## Introduction

Apoptosis-associated speck-like protein containing a CARD (ASC) was originally identified as a protein encoded by the Pycard gene (1–3). ASC has been widely studied in innate immune cells (macrophages, dendritic cells, and neutrophils) as an adaptor molecule of the multiprotein signaling complex known as the inflammasome. The inflammasome has various functions, including the proteolytic cleavage of the immature pro-inflammatory cytokines IL-1β and IL-18, and cell death by pyroptosis (4). ASC is central to the formation of different inflammasome complexes comprising the NLRP1 (5), NLRP3 (6, 7), NLRP6 (8, 9), AIM2 (10), and NLRC4 (11–13) sensor molecules. Upon sensing specific triggers, these sensor molecules undergo conformational changes that catalyze ASC oligomerization to form a macromolecular signaling platform known as the ASC “speck.” ASC then recruits the cysteine protease caspase-1 via its C-terminal CARD domain (14–16).

Crohn's disease and ulcerative colitis are chronic inflammatory bowel diseases (IBDs) that affect the gastrointestinal tract (17). The pathogenic inflammation in IBD is characterized by an imbalance in mucosal immunity in the intestine triggered by genetic and environmental factors. This imbalance leads to hyperactivation of innate and adaptive immune and non-immune processes against self-antigens in the gut leading to severe chronic immunopathology. The mechanisms by which IBDs develop and progress are complex and still largely unknown.

Recent studies have found that various inflammasomes, particularly the NLRP3 inflammasome, help regulate gut homeostasis in a complicated process that is dependent on the presence and composition of the intestinal microbiome (18). These inflammasomes are also associated with the immunopathology underlying chronic intestinal inflammation (18–20). Asc^−/−^ mice develop markedly more severe pathology after dextran sodium sulfate (DSS)-induced colitis than wild-type (WT) and Casp1^−/−^ mice (21). The protective role of ASC is mainly attributed to its inflammasome function in myeloid and intestinal epithelial cells, which by secreting the cytoprotective IL-18 cytokine, helps ensure appropriate intestinal epithelial cell proliferation, tissue repair upon wounding, and anti-microbial defenses (18).

Many experimental studies using rodent models have revealed that chronic colitis and intestinal inflammation results from a dysregulated immune response primarily derived from perturbed T-cell homeostasis. Only a few studies, however, have addressed the contribution of ASC in CD4^+^ T-cell responses. Thus, the intrinsic role of ASC in T cells, particularly in the context of intestinal inflammation, remains uncharacterized. Narayan et al. reported that Asc^−/−^ CD4^+^ T cells produce higher levels of the immunosuppressive cytokine IL-10 and lower levels of IL-2 (22). This effect leads to an expansion of Foxp3^−^ T regulatory (Treg) cells and suppression of bystander T-cell proliferation. Another study has suggested that mature Asc^−/−^ CD4^+^ T cells show reduced survival in the periphery when co-transferred with WT T cells into naïve mice (23). A recent study proposed that T-cell intrinsic ASC has, in part, a protective role in a mouse model of experimental autoimmune encephalomyelitis, as T helper 17 (Th17)-mediated neuroinflammation was less evident in mice with a T-cell-specific deletion of ASC compared to WT mice (24).

Here, we investigated the T-cell intrinsic contribution of ASC in immune homeostasis in the intestinal tract. We found that Asc^−/−^, but not Nlrp3^−/−^ and Casp-1^−/−^, CD4^+^ T cells exacerbate T-cell induced colitis, suggesting that ASC in CD4^+^ T cells has a protective role. Asc^−/−^ CD4^+^ T cells possess a higher proliferative capacity *in vitro* and *in vivo* and displayed robust TCR-mediated activation and inflammatory activity compared to WT cells. These findings demonstrate that ASC shapes adaptive immunity independently of inflammasomes, by modulating cell-intrinsic activation and proliferation.

## Results

### Asc^−/−^ CD4^+^ T Cells Exhibit Enhanced Spontaneous Activation *in vivo*

**Figure 1:**
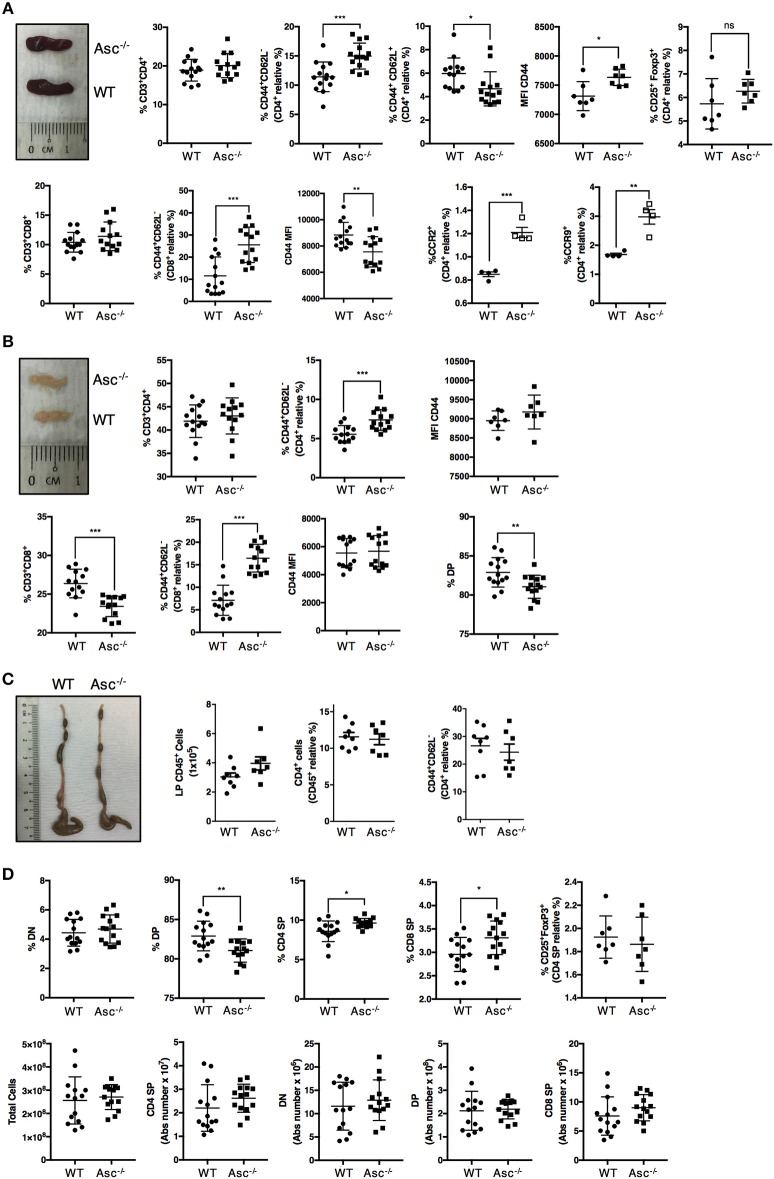
CD4^+^ T cells have an activated phenotype in steady state in Asc^−/−^ mice. Proportions of total CD3^+^/CD4^+^, CD3^+^/CD8^+^, and effector (CD44^+^CD62L^−^), central memory (CD62L^+^CD44^+^) cells, and mean fluorescence intensity (MFI) of CD44 expression and percentage of CD25^+^FoxP3^+^ Treg cells in the spleens **(A)** and mesenteric lymph nodes **(B)** of wild-type (WT) and Asc^−/−^ mice **(C)**. The number of CD45^+^ cells, and percentage of total CD4^+^ and CD4^+^ expressing the CD44 marker in colonic lamina propria (LP) cells. Representative images of the spleen **(A)**, mesenteric lymph node (LN) **(B)**, and colon **(C)** of WT and Asc^−/−^ mice at 8–10 weeks of age are also shown. **(D)** Immune phenotype of the thymus of 8–10 weeks old WT and Asc^−/−^ mice. All data represent the means ± standard error of three experiments (*n* = 4–5 mice/group per experiment). *P < 0.05, **P < 0.01, ***P < 0.001.

To date, only a few studies have aimed to characterize the T-cell compartment of Asc^−/−^ mice. The spleen and mesenteric lymph nodes (mLNs) of 8–12-week-old Asc^−/−^ mice were structurally normal and showed a normal frequency of total CD4^+^ T cells (Figures 1A,B; Supplementary Figures 1a,b). However, a higher proportion of CD4^+^ T cells expressing the activation marker CD44 was detected in both the spleen and mLN of Asc^−/−^ mice compared to age-matched WT mice (Figures 1A,B; Supplementary Figures 1a,b). Splenic and mLN CD44^+^ CD4^+^ T cells of Asc^−/−^ mice also expressed CD44 at higher levels, as determined by mean fluorescence intensity (Figures 1A,B), indicating an increased antigen-experienced phenotype of CD4^+^ T cells lacking ASC. We also detected a higher proportion of splenic CD4^+^ T cells expressing the chemokine receptors CCR2 and CCR9 in Asc^−/−^ mice compared to WT mice (Figure 1A). Steady-state basal activation of CD4^+^ T cells is maintained throughout the murine life-span, as we observed this phenotype in both young (6 weeks) and old (40 weeks) mice (data not shown). Moreover, the substantial increase in activated T cells observed under steady-state conditions in Asc^−/−^ mice was replicated in the CD8^+^ T-cell compartment of the spleen and mLN of these mice. However, the level of CD44 expression on Asc^−/−^ CD8^+^ T cells was lower than WT in spleen and remained unchanged in mLN (Figures 1A,B; Supplementary Figures 1a,b). Despite this evident T-cell activation, no obvious immunopathology, such as splenomegaly or lymphadenopathy, was detected in Asc^−/−^ mice compared to WT mice. Macroscopical examination of the colon and phenotypical characterization of CD44^+^ CD4^+^ T cells from the colonic LP of Asc^−/−^ mice showed no abnormalities (Figure 1C).

To determine whether sustained T-cell activation occurred during T-cell development, we examined the different T-cell populations in the thymus of 6–8 weeks old mice. The percentage of the double positive (DP) CD4^+^CD8^+^ population was slightly reduced, and the CD4 and CD8 single positive (SP) fractions were slightly elevated in the thymus of Asc^−/−^ mice compared to age-matched WT mice (Figure 1D; Supplementary Figure 1c). These differences, however, were not reflected by the absolute numbers as the total number of DP, CD4 SP, and CD8 SP was not significantly altered in the thymus of Asc^−/−^ mice compared to WT mice (Figure 1D).

### ASC, NLRP3, and Caspase-1 Are Expressed in Naïve and Activated CD4^+^ T Cells

**Figure 2:**
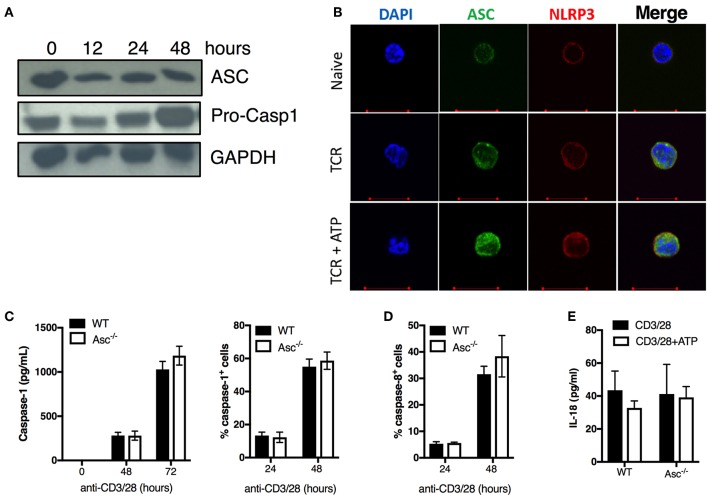
ASC expression, caspase 1/8 activation, and IL-18 release in naïve and activated CD4^+^ T cells. **(A)** Immunoblot analysis of ASC and pro-caspase-1 in wild-type (WT) naïve and anti-CD3/CD28 activated CD4^+^ T cells at the indicated times. **(B)** Confocal analysis of ASC and NLRP3 expression in naïve CD4^+^ T cells stimulated with anti-CD3/CD28 for 72 h with or without additional stimulation for 8 h with the inflammasome activator ATP. **(C)** Caspase-1 and **(D)** caspase-8 activation assessed by FAM-FLICA assay in WT and Asc^−/−^ CD4^+^ T cells at 24 and 48 h post-stimulation with anti-CD3/CD28 antibodies. **(C)** Caspase-1 release in the supernatants from anti-CD3/CD28 activated WT and Asc^−/−^ CD4^+^ T cells was measured by ELISA at 48 and 72 h post-stimulation with anti-CD3/CD28 antibodies. **(E)** Levels of IL-18 release by WT and Asc^−/−^ CD4^+^ T cells 72 h post-activation with anti-CD3/CD28 antibodies with or without additional 8 h exposure to ATP. All data represent the means ± standard error of representative experiments (*n* = 3).

To examine the effect of ASC depletion in CD4^+^ T cells, we first assessed ASC protein expression at basal level and upon stimulation via TCR triggering. ASC was highly expressed in naïve CD4^+^ T cells and was widely maintained up to 48 h post-activation (Figure 2A). ASC localization was also assessed by confocal immunofluorescence microscopy. In naïve cells, ASC showed a diffuse cytoplasmic/nuclear localization; upon TCR activation ASC signal was more evident due to cytosol enlargement (Figure 2B). TCR activation, in combination with ATP stimulation (to activate the NLRP3 inflammasome), did not substantially alter the ASC localization profile in CD4^+^ T cells from TCR stimulation alone (Figure 2B). We also analyzed the expression of the inflammasome sensor NLRP3. CD4^+^ T cells expressed NLRP3 in both steady state conditions and upon TCR triggering, showing a similar localization pattern as ASC in the presence or absence of ATP (Figure 2B). We observed dotted ASC-containing structures in TCR-activated CD4^+^ T cells, which disappeared upon ATP stimulation. These did not look similar to the typical ASC-speck structures that are commonly visible in macrophages, upon inflammasome activation (25) (Figure 2B).

We also examined the expression of the caspase-1 precursor (pro-casp-1) and its activation state in unstimulated and TCR-activated CD4^+^ T cells. Pro-casp-1 was expressed at steady state and was increased upon CD4^+^ T-cell activation with anti-CD3/CD28 antibodies (Figure 2A), suggesting that casp-1 activation may also occur in these cells. Although we could not detect the cleaved form of caspase-1 by western blot, we found that casp-1 activation and release were induced upon TCR stimulation in CD4^+^ T cells in a time-dependent manner by using cytofluorimetric and ELISA assays (Figure 2C). Casp-1 activation and secretion occurred independently of ASC as the levels were similar between WT and Asc^−/−^ CD4^+^ T cells (Figure 2C). Similarly, casp-8 activation, which is an executioner caspase involved in apoptosis (24), occurred regularly in Asc^−/−^ CD4^+^ T cells (Figure 2D). Moreover, we only detected low levels of IL-18 produced by CD4^+^ T cells following TCR stimulation (Figure 2E). Again, IL-18 release was independent of ASC as we could not identify any differences in IL-18 levels between WT and Asc^−/−^ T cells (Figure 2E). No measurable IL-1β was detected in supernatants of naïve CD4^+^ T cells stimulated with anti-CD3/28 antibodies alone or in combination with ATP (data not shown).

In summary, we did not observe a typical pattern of classic inflammasome activation in CD4^+^ T cells, including ASC speck formation, IL-1β/IL-18 release, and casp-1 activation dependently on ASC. These findings suggest that these cells do not assemble a functional NLRP3 inflammasome to release notable levels of bioactive IL-1β and IL-18.

### ASC Deficiency in CD4^+^ T Cells Exacerbates Colitis in an Adoptive Transfer T-Cell-Induced Colitis Mouse Model

Most of the current literature on ASC function in intestinal inflammation has been linked to its prominent role in the inflammasomes that assemble in innate immune cells and intestinal epithelial cells. Furthermore, acute epithelial injury models, such as the most frequently used DSS-induced colitis model, have typically been used (26). However, DSS-induced colitis can even develop in T-cell deficient mice (27), indicating that T and B cells are not required for colitis development in this model. Thus, it has been impossible to determine the role of ASC in the context of T-cell dependent colitis, an essential component of IBD. Moreover, not much was known about the biological significance of ASC in lymphocytes, in particular, CD4^+^ T cells, and its contribution to the pathogenesis of inflammation-related autoimmune colitis. Thus, we utilized a T cell transfer model of colitis to investigate whether ASC has an intrinsic role in CD4^+^ T cells in intestinal inflammation.

**Figure 3:**
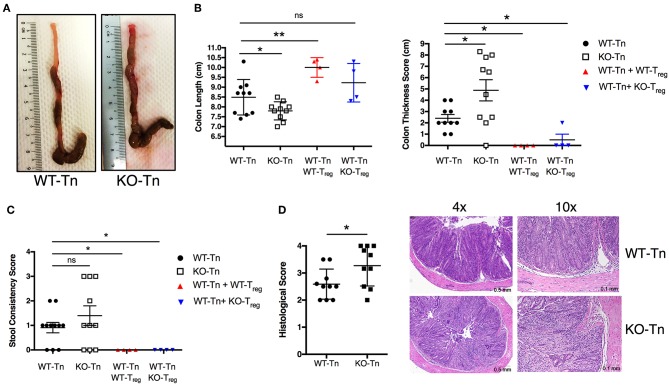
Lack of ASC expression in CD4^+^ T cells augments the colitogenic activity of these cells when transferred *in vivo*. Sorted splenic naïve CD4^+^ T cells (CD45RB^high^CD62L^+^CD44^−^CD25^−^) from wild-type (WT) or Asc^−/−^ mice (3 × 10^5^ cells) were adoptively transferred (with or without 10^5^ sorted CD4^+^CD25^high^ Treg cells) into Rag1^−/−^ mice. Mice were sacrificed at 15 weeks post-transfer, and the colons were analyzed for macroscopic and microscopic signs of pathology. **(A)** Representative images of the colon in WT or Asc^−/−^ mice 15 weeks after T-cell transfer. **(B)** Colon length and thickness score and **(C)** stool consistency score. **(D)** Histological score of the colon tissues, with representative images of H&E stained sections. Tn, T Naïve only. All data represent the means ± standard error of three experiments (*n* = 3–6 mice/group per experiment). *P < 0.05, **P < 0.01.

To decipher in depth the role of ASC specifically in CD4^+^ T cells *in vivo*, we adoptively transferred Asc^−/−^ and WT naïve CD4^+^ T cells into Rag1^−/−^ mice, and examined pathology and T-cell expansion in the colonic LP. Although the two groups lost weight at a similar rate (data not shown), Rag1^−/−^ mice receiving Asc^−/−^ CD4^+^ T cells showed exacerbated signs of pathology and colonic inflammation at 100 days post transfer, including shortening and thickening of the colon and loose stools/diarrhea, compared to Rag1^−/−^ mice receiving WT CD4^+^ T cells (Figures 3A–C). The histological score was significantly higher in Rag1^−/−^ mice adoptively transferred with Asc^−/−^ CD4^+^ T cells compared to Rag1^−/−^ mice receiving WT CD4^+^ T cells (Figure 3D). An in-depth histological examination revealed a more severe transmural gut inflammation in Rag1^−/−^ mice receiving Asc^−/−^ naïve CD4+ T cells, with massive infiltration of inflammatory cells into the colon and crypt abscesses compared to WT T naïve mice (Figure 3D).

We next co-transferred WT or Asc^−/−^ regulatory T cells (Treg) with WT CD4^+^ T cells in Rag1^−/−^ mice. WT and Asc^−/−^ Tregs equally restored colon length and thickness, and normalized stool consistency in mice also receiving WT CD4^+^ T cells, indicating that Asc^−/−^ Treg cells are functional *in vivo* and can inhibit WT T-cell effector functions therefore preventing the onset of colitis (Figures 3B,C).

**Figure 4:**
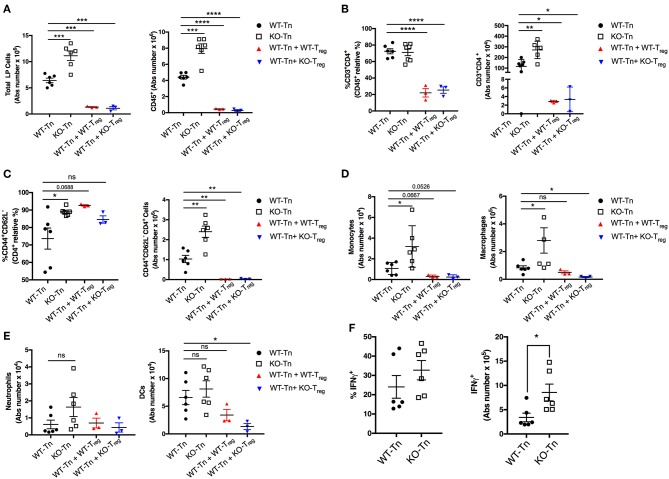
ASC suppresses pathogenic CD4^+^ T-cell expansion in the colon. **(A)** Total number of colonic LP cells, number of CD45^+^ cells, **(B,C)** percentage and numbers of total and effector (CD44^+^CD62L^−^) CD4^+^ cells, and **(D,E)** number of monocytes (CD45^+^F4/80^+^CD11b^+^Ly6C^+^), macrophages (CD45^+^F4/80^+^CD11b^+^Ly6C^−^Ly6G^−^), neutrophils (CD45^+^F4/80^−^CD11b^+^Ly6C^+^Ly6G^hi^), and dendritic cells (CD45^+^CD11c^hi^MHCII^hi^) in lamina propria (LP) cells. **(F)** LP cells were re-stimulated *ex vivo* with PMA (500 ng/mL) and ionomycin (1 μg/mL) for 5 h (Brefeldin A was added for the last 4 h) and stained intracellularly for IFNγ detection in CD4^+^ T cells. Tn T Naïve only. All data represent the means ± standard error of 2–3 experiments (*n* = 3-6 mice/group per experiment). *P < 0.05, **P < 0.01, ***P < 0.001, ****P < 0.0001.

Flow cytometric analysis showed a higher number of total and CD45^+^ immune cells recovered from the colonic LP in the mice receiving Asc^−/−^ CD4^+^ T cells (Figures 4A,B). The proportion and number of CD44^+^ CD4^+^ effector T cells were also higher in the intestinal LP, together with a higher level of monocytic infiltrate (Figure 4C,D), while other myeloid subsets were not significantly different (Figure 4E). There was also a higher number of colitogenic Asc^−/−^ CD4^+^ T cells producing IFNγ compared to WT cells (Figure 4F). These data suggest an anti-inflammatory and protective role of ASC in CD4^+^ T cells in the gut.

To examine whether the inflammasome sensor NLRP3 may also be involved in this process, we evaluated the course of colitis in Rag1^−/−^ mice receiving naïve CD4^+^ T cells sorted from the spleen of Nlrp3^−/−^ mice. These mice developed a mild form of colitis comparable to that of Rag1^−/−^ mice adoptively transferred with WT CD4^+^ T cells (Supplementary Figure 2a). Likewise, the transfer of Casp-1^−/−^ naïve CD4^+^ T cells induced a colitic phenotype similar to mice receiving WT CD4^+^ T cells (Supplementary Figure 2b). These data illustrate that lack of NLRP3 or Casp-1 expression in CD4^+^ T cells does not exacerbate colitis in these transfer models, pointing to an inflammasome-independent protective function of ASC in CD4^+^ T cells.

### ASC Is Dispensable for CD4^+^ T-Helper Cell Lineage Differentiation

**Figure 5:**
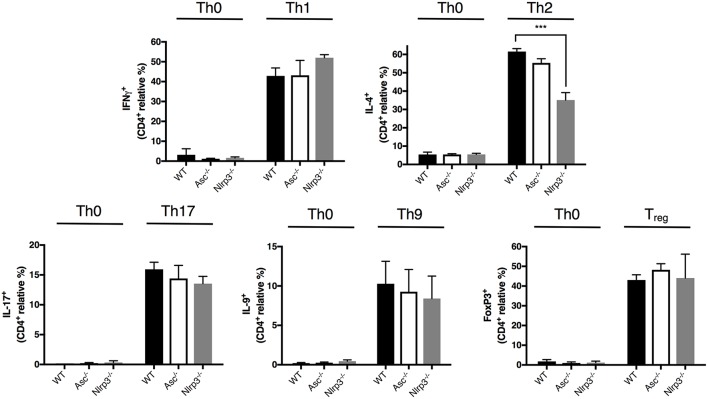
Lack of ASC does not affect CD4^+^ T-cell polarization into different T helper and Treg cell fates. Naïve CD4^+^ T cells (CD62L^+^CD44^−^CD25^−^) were sorted from the spleens of WT, Asc^−/−^, and Nlrp3^−/−^ mice and polarized into Th1, Th2, Th17, Th9, and Treg lineages. Cells were then fixed and stained intracellularly for representative cytokines for each T helper lineage and Foxp3 expression, and analyzed by flow cytometry. All data represent the means ± standard error of three experiments. ***P < 0.001.

CD4^+^ T cells exert their specialized functions in different contexts by polarizing into T helper (Th) subsets based on interactions with other immune cells and cytokines. The high level of T-cell activation in Asc^−/−^ mice led us to examine the possibility that ASC may be involved in CD4^+^ T cell functional polarization. We differentiated *in vitro* naïve CD4^+^ T cells from Asc^−/−^ and WT mice into Th1, Th2, Th17, Th9, and Treg lineages using standard culture conditions. The polarization of Asc^−/−^ CD4^+^ T cells toward all these subsets *in vitro* was indistinguishable from that of WT CD4^+^ T cells (Figure 5). Nlrp3^−/−^ naïve T cells exhibited defective Th2 polarization, as previously reported (28), while all other Th subsets normally differentiated (Figure 5). These data indicate that loss of ASC does not impair the ability of naïve T cells to polarize into various Th subsets when stimulated *in vitro* with a particular cytokine milieu. However, we cannot exclude the possibility that these cells might behave differently and exhibit a perturbed T-cell polarization profile *in vivo*.

### Lack of Intrinsic ASC Expression Enhances CD4^+^ T-Cell Proliferation

**Figure 6:**
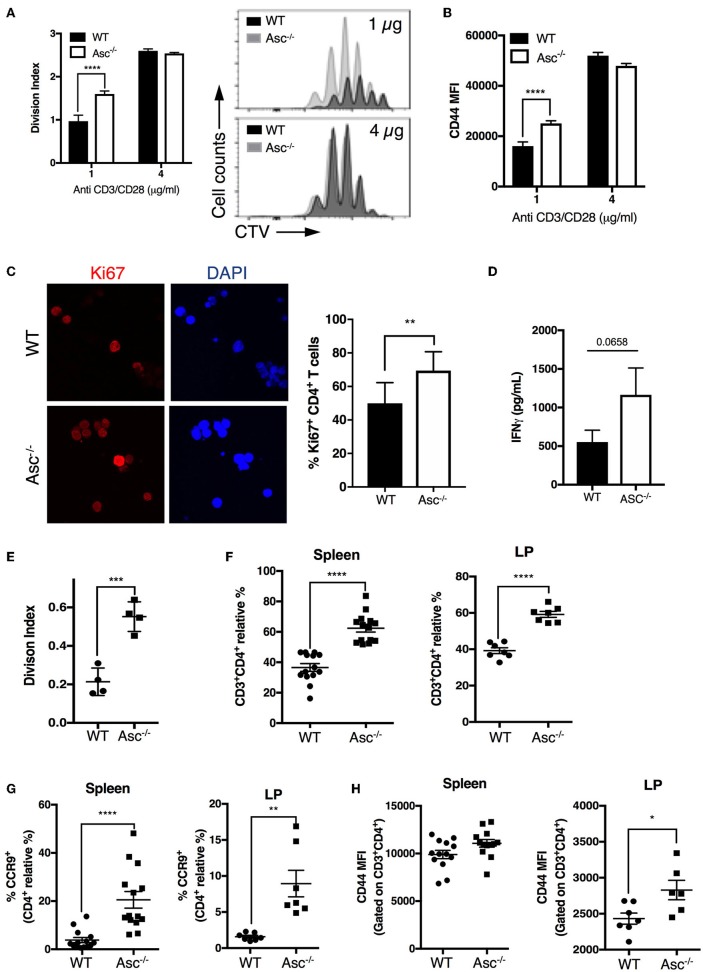
ASC-deficient CD4^+^ T cells proliferate at a higher rate than WT cells *in vitro* and *in vivo*. **(A,B)** Splenic wild-type (WT) and Asc^−/−^ naïve CD4^+^ T cells were stained with CellTrace Violet (CTV) dye and stimulated with anti-CD3/CD28 antibodies for 72 h, and CTV dilution was analyzed by flow cytometry. Division index **(A)** and mean fluorescence intensity (MFI) of CD44 expression **(B)** are shown. **(C)** Confocal analysis of Ki-67^+^ proliferating WT and Asc^−/−^ naïve CD4^+^ T cells stimulated with anti-CD3 (5 μg/ml) and anti-CD28 (2 μg/ml) antibodies for 48 h. Representative images of Ki-67 labeling are shown. DAPI, blue; Ki-67, red. Scale bar 20 μm. **(D)** IFNγ production in the supernatants of naïve WT and Asc^−/−^ CD4^+^ T cells stimulated with anti-CD3 (5 μg/ml) and anti-CD28 (2 μg/ml) antibodies for 72 h. **(E)** Purified splenic naïve CD4^+^ T cells (1.5 × 10^6^) from WT and Asc^−/−^ mice were stained with CTV and adoptively transferred into Rag1^−/−^ mice. Spleens were harvested and CTV dilution was analyzed in CD4^+^ T cells by flow cytometry 4 days after transfer. The division index is shown. **(F–H)** Sorted splenic CD4^+^ T cells (CD45RB^high^CD44^−^CD62L^+^CD25^−^) from WT and Asc^−/−^ mice were adoptively co-transferred at a 50:50 ratio into Rag1^−/−^ recipients. Mice were sacrificed, and spleens and colonic LP cells were analyzed by flow cytometry. Percentages of WT and Asc^−/−^ CD3^+^CD4^+^ cells **(F)**, CCR9^+^ CD4^+^ T cells **(G)**, and MFI of CD44 expression on these cells **(H)** are shown. All data represent the means ± standard error of two experiments (*n* = 7 mice/group per experiment). *P < 0.05, **P < 0.01, ***P < 0.001, ****P < 0.0001.

As CD4^+^ T cells lacking ASC showed a higher basal activation level in the steady state *in vivo*, we examined the possibility that these cells also possess a superior proliferative capacity compared to WT T cells. Tracking T-cell proliferation by Cell Trace Violet (CTV)-labeling showed that Asc^−/−^ CD4^+^ T cells indeed have a higher proliferation rate (measured in terms of the division index) and CD44 expression level when activated with a low dose (1 μg/ml) of anti-CD3/CD28 antibodies, while this difference was not present at high dose anti-CD3/CD28 (4 μg/ml) (Figures 6A,B). This higher capacity for Asc^−/−^ CD4^+^ T cells to proliferate compared to WT T cells in response to TCR triggering was also confirmed by monitoring the intracellular expression of Ki-67 that is expressed by cycling or recently divided cells (Figure 6C). Although not significantly, Asc^−/−^ CD4^+^ T cells also tended to release more IFNγ (Figure 6D), which is indicative of both an activated phenotype and high proliferative capacity.

To examine whether loss of ASC triggers robust CD4^+^ T-cell expansion *in vivo*, we adoptively transferred CTV-labeled Asc^−/−^ and WT naïve CD4^+^ T cells into immunodeficient Rag1^−/−^ host mice and assessed T-cell proliferation in the spleen 4 days after transfer by flow cytometry. Asc^−/−^ CD4^+^ T cells had a significantly higher division index compared to WT cells (Figure 6E). In addition, Asc^−/−^ CD4^+^ T cells outcompeted WT CD4^+^ T-cell expansion when adoptively transferred at a 50:50 ratio in immunodeficient host mice: a higher proportion of Asc^−/−^ total and the gut-homing CCR9^+^ CD4^+^ T cells were recovered from the spleens and colonic LP of Rag1^−/−^ mice 50 days post transfer compared to WT CD4^+^ T cells (Figures 6F,G). Moreover, Asc^−/−^ CD4^+^ T cells from spleen and colonic LP expressed relatively higher levels of the CD44 marker, suggesting that they appear to be slightly more activated (Figure 6H).

### ASC-Deficient CD4^+^ T Cells Possess a Robust Signaling and Metabolic Profile

**Figure 7:**
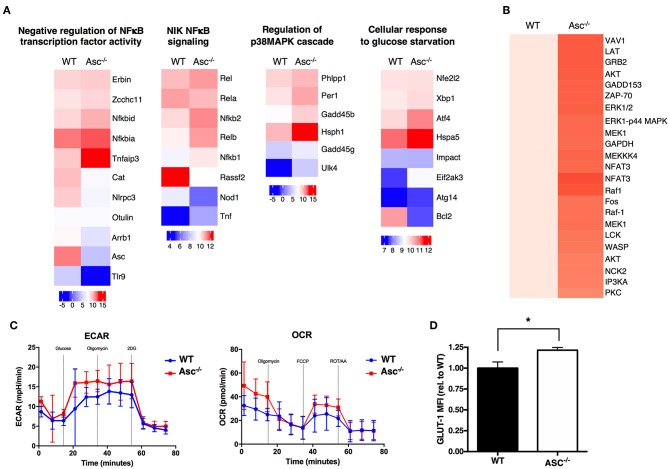
ASC-deficient CD4^+^ T cells have a more pro-inflammatory profile, robust TCR-triggered signature, and are metabolically active than WT cells. **(A)** Wild-type (WT) and Asc^−/−^ sorted naïve CD4^+^ T cells were left untreated or stimulated with anti-CD3 (5 μg/ml) and anti-CD28 (2 μg/ml) antibodies for 12 h and then analyzed by RNA-Sequencing. Graphs show the relative expression of selected genes. **(B)** Purified splenic WT and Asc^−/−^ naïve CD4^+^ T cells were stimulated with anti-CD3/CD28 antibodies, and the phosphorylation profile of the TCR-related proteins was assessed using a TCR phosphoprotein array. **(C)** Glycolytic (ECAR) and mitochondrial (OCR) stress profiles of WT and Asc^−/−^ sorted naïve CD4^+^ T cells 16 h post-activation with anti-CD3/CD28 antibodies. **(D)** WT and Asc^−/−^ naïve CD4^+^ T cells were activated for 48 h with anti-CD3/CD28 antibodies, and intracellular GLUT-1 expression was measured by flow cytometry and expressed as the mean fluorescence intensity (MFI) relative to WT. All data represent the means ± standard error of a representative experiment (*n* = 5 mice). *P < 0.05.

To understand the possible mechanisms driving the hyperactive Asc^−/−^ CD4^+^ T-cell profile, we analyzed the global gene expression of these cells under steady-state condition and upon stimulation with anti-CD3/CD28 antibodies *in vitro* by RNA-sequencing. Pro-inflammatory genes including Nfkb1, NFKb2, Rel, and Relb, as well as some members of p38 MAPK cascade showed a higher expression in Asc^−/−^ CD4^+^ T cells particularly at post-activation, compared to WT cells, which is suggestive of a more activated and pro-inflammatory signature (Figure 7A).

A phosphoprotein analysis of the proteins involved in TCR signaling in CD4^+^ T cells activated by anti-CD3/CD28 antibodies revealed that several proteins were differentially expressed or activated in Asc^−/−^ CD4^+^ T cells compared to WT T cells (Figure 7B). Many of these proteins are involved in TCR signaling and T-cell activation, which confirm a more robust T-cell activation in the absence of Asc compared to the WT controls.

Tight metabolic regulation ensures that activated T cells meet the increased energy requirements for activation and proliferation. We thus assessed the metabolic profile of the Asc^−/−^ CD4^+^ T cells upon *in vitro* stimulation with anti-CD3/CD28 antibodies for 16 h. We found that Asc^−/−^ CD4^+^ T cells showed higher basal levels of glycolysis, which increased in TCR-activated cells, suggesting an augmented metabolically active state when compared to WT CD4^+^ T cells (Figure 7C). Higher glycolytic flux in Asc^−/−^ CD4^+^ T cells compared to WT T cells was consistent with a higher level of expression of Glut-1, the most widely expressed glucose transporter (Figure 7D).

In conclusion, these findings suggest that ASC controls CD4^+^ T-cell biology at multiple stages, including TCR signaling, activation, pro-inflammatory responses, and metabolic activities.

## Discussion

Most of the current knowledge on ASC function has been linked to its prominent role in the assembly of the inflammasomes. While ASC has been mostly studied in innate immune cells, the cellular functions governed by ASC in other immune cells remain to be investigated. This study uncovers a new role of ASC in T helper cells in regulating gut pathology. We found that when adoptively transferred into Rag1^−/−^ mice, naïve Asc^−/−^ CD4^+^ T cells were more aggressive and colitogenic *in vivo* than WT CD4^+^ T cells. Mice receiving Asc^−/−^ CD4^+^ T cells had more severe colitis as highlighted by higher clinical and histological scores, and a markedly higher number of activated T cells infiltrating the colonic LP. Conversely, loss of Nlrp3 or Caspase-1 expression in CD4^+^ T cells did not confer a highly colitogenic phenotype *in vivo* in a T-cell adoptive transfer colitis model. In addition, the phenotype of disease in Rag1^−/−^ mice receiving Nlrp3^−/−^ or Casp-1^−/−^ CD4^+^ T cells did not resemble the exacerbated colitis observed in mice receiving Asc^−/−^ CD4^+^ T cells. This finding suggests that it is unlikely that these inflammasome components are involved in ASC-mediated regulation of CD4^+^ T-cell function. However, we cannot rule out the possibility of ASC engaging other receptors such as AIM2 or NLRP6 to form a functional inflammasome complex in CD4^+^ T cells.

We confirmed that ASC is constitutively expressed in naïve and CD3/CD28 activated CD4^+^ T cells, and confocal analysis showed that its expression increased upon TCR triggering. Martin et al. have previously shown trace amounts of IL-1β released by CD3/CD28 activated CD4^+^ T cells (24). Although NLRP3, caspase-1 and IL-1β precursors are expressed in naïve and activated CD4^+^ T cells, we did not detect IL-1β release at a measurable level after TCR triggering and ATP treatment, questioning of a potential role of T-cell-derived IL-1β. In addition, we detected very low levels of IL-18 released by activated CD4^+^ T cells, which might correspond to the IL-18 precursor; this release was independent of ASC expression. A recent study has reported that IL-18Rα-deficient CD4^+^ T cells induced comparable intestinal pathology to WT CD4^+^T cells, concluding that IL-18-dependent cytokine induced activation of these cells is not critical for the development of T-cell-induced colitis (29).

Insights into a possible T-cell intrinsic mechanism underlying exacerbated colitis came from our initial observation that CD4^+^ Th cells and CD8^+^ cytotoxic T cells in Asc^−/−^ mice exhibited a significantly more activated phenotype than in WT mice. Despite this phenotype, the mice did not develop any spontaneous inflammation, suggesting that the hyperactivity of these cells in the full knock out mice is somehow controlled under pathogen-free conditions. The higher percentage of CD44^+^ effector T cells in spleens and lymph nodes of these mice suggested a cell-intrinsic defect in the regulation of T-cell activation in the absence of ASC. We observed that Asc^−/−^ CD4^+^ T cells expanded at a faster pace when stimulated *in vitro* by CD3/CD28 antibodies and tended to release higher levels of IFNγ compared to WT CD4^+^ T cells. This difference was more evident when we stimulated Asc^−/−^ CD4^+^ T cells with a sub-optimal, low dose of anti CD3/CD28 antibodies, suggesting that ASC exerts its regulatory effect on T-cell activation/proliferation depending on the threshold of the TCR signal received. We also observed a higher percentage of Asc^−/−^ CD4^+^ T cells expressing Ki-67 (a commonly used cell proliferation marker) compared to WT T cells. Consistently, Asc^−/−^ CD4^+^ T cells exhibited more robust homeostatic proliferation when adoptively transferred into immunocompromised mice than WT naïve CD4^+^ T cells. Using a co-transfer paradigm, we found that Asc^−/−^ CD4^+^ T cells out-competed WT naïve CD4^+^ T cells and populated the spleen, lymph nodes, and the colonic LP in Rag1^−/−^ mice as the dominant population. Loss of ASC altered the proliferative, but not the functional capacity of naïve CD4^+^ T cells, as these cells showed normal polarization toward all known helper populations (Th1, Th2, Th17, and Th9) and Tregs, at least *in vitro*.

We have also shown that Asc^−/−^ Tregs have no defect in their suppressive capacity *in vivo* as they were able to restrain the proliferation of WT CD4^+^ T cells in our colitis model. However, an important question remaining to be answered is whether the Asc^−/−^ Tregs are also potent enough to prevent the onset of exacerbated colitis caused by adoptive transfer Asc^−/−^ naïve CD4^+^ T cells into Rag-1^−/−^ recipients.

The current literature on T-cell proliferation in the absence of ASC expression is rather limited. Narayan et al. reported that activated Asc^−/−^ CD4^+^ T cells produced high amounts of IL-10 and released low amounts of IL-2, therefore indirectly suppressed effector T-cell proliferation *in vitro* (22). We did not find any significant difference in IL-10 levels produced between WT and Asc^−/−^ CD4^+^ T cells following stimulation with anti-CD3/CD28 antibodies (data not shown). Another group showed a normal antigen-specific T-cell response by Asc^−/−^ T cells and comparable T-cell proliferation between WT and Asc^−/−^ T cells after TCR triggering *in vitro* (30). By this experimental model, the identity of the cells and the hosts have differed. Moreover, no report has addressed the ASC requirement in CD4^+^ T cells during colitis, and thus, our results are not comparable.

The precise mechanism on how ASC regulates T-cell proliferation remains to be determined. The anti-proliferative nature of ASC (previously known as TMS-1) has also been linked to cancer progression (4). Indeed, it was found that the ASC promoter is highly methylated in several cancers, which silences ASC tumor-suppressor activity and permits cancer-cell propagation (31–36). Some other studies more broadly have implicated the NLRP3 inflammasome in cancer and links to proliferation of T cells/lymphoma cells. In multiple myeloma patients with a mutation in CARD8 (a negative regulator of NLRP3 inflammasome) had a significantly higher percentage of pro-inflammatory Th1 cells (37). Another study from the same group has shown that NLRP3 inflammasome activation promoted lymphoma cells proliferation and inhibited apoptosis through up-regulation of c-myc and bcl-2 anti-apoptotic factors (38).

Recents studies have shown that the interaction of NEK7, a member of the family of mammalian NIMA-related kinases, with NLRP3 is required for NLRP3 oligomerization and subsequent activation of the inflammasome (39–41). This process was shown to be dependent on potassium efflux (39). NEK7 plays a crucial role in the progression of the cell cycle and particularly during G1, by regulating mitotic spindle organization and centriole duplication (40). It was shown that NEK7 could act as a cellular switch to exert mutual exclusivity of the inflammasome responses and cells division (40). It is, therefore, possible that in proliferating cells, like T cells, most of the NEK7 proteins are involved in the regulation of cell cycle and the residual proteins are not sufficient to induce the formation of the inflammasome complex. In contrast, in cells of the myeloid lineage (i.e., macrophages, dendritic cells), which have a low proliferating capacity, most of the NEK7 proteins could be able to bind to NLRP3 leading to the assembly of a functional NLRP3 inflammasome. It is of great interest to study the role of NEK7 in the regulation of ASC function in CD4^+^ T cells in future studies to further dissect this possible mechanism.

ASC has also been shown to modulate the NF-κB signaling by suppressing components of the IKK complex and interacting with PAAD-only protein-1 (POP1)/ASC2 protein (42, 43). These events lead to NF-κB inhibition, which has a broad role in inflammation and cancer. Indeed, we found that genes associated with the NF-κB pathway were increased in Asc^−/−^ CD4^+^ T cells compared to WT T cells upon TCR stimulation.

Finally, T-cell activation and proliferation are tightly controlled by the rate of energy consumption and various metabolic pathways. We thus assessed the metabolic profile of activated CD4^+^ T cells in the absence of ASC expression. We found that Asc^−/−^ T cells manifest an elevated rate of glycolysis, corroborating the highly active, and proliferative nature of these cells compared to WT cells.

Collectively, we have identified that ASC regulates CD4^+^ T-cell activation state by partially restraining elevated metabolic rates, and ultimately inhibiting uncontrolled expansion of these cells mainly in the gut mucosa. Further studies are needed to investigate the importance of ASC in other T-cell mediated autoimmune diseases, as well as cancer models of T-cell leukemia and lymphoma.

## Materials and Methods

### Mice

C57BL/6J and Rag1^−/−^ mice were purchased from the Biological Resource Center (Agency for Science, Technology and Research, A^*^STAR, Singapore). B6.SJL-PtprcaPepcb/BoyJ (CD45.1^+^) mice were purchased from The Jackson Laboratory (USA). Nlrp3^tm1Tsc^ (Nlrp3^−/−^) mice were provided by the University of Lausanne (Switzerland), Pycard^tm1Vmd^ (Asc^−/−^) and Caspase1^−/−^ (Casp-1^−/−^) mice were obtained from V. M. Dixit (Genentech). Age-matched and gender-matched wild-type mice were used in all experiments. All animals were congenic on a C57BL/6J background and were maintained at the Biological Resource Center (A^*^STAR) under specific pathogen-free conditions. All experimental procedures were approved by the Institutional Animal Care and Use Committee (IACUC) of the Biological Resource Center (BRC), A^*^STAR in compliance with their Guidelines for Animal Experiments.

### T-Cell Isolation, Stimulation, and *in vitro* Proliferation Assay

Naïve CD4^+^ T cells were isolated from mouse spleens using an EasySepTM Mouse Naïve CD4^+^ T Cell isolation kit (Stem Cell Technologies), following the manufacturer's instructions. Cells were seeded in flat-bottom cell-culture treated 96-well plates (Corning) at a density of 10^5^ cells/well and cultured in complete RPMI 1640 medium with L-Glutamine (2 mM) supplemented with 10% fetal bovine serum (FBS), penicillin (100 IU/ml), streptomycin (100 μg/ml) (all from GIBCO). Cells were stimulated with plate-bound anti-CD3 (5 μg/ml) and soluble anti-CD28 (2 μg/ml) in the presence of recombinant mouse IL-2 (20 ng/ml; R&D Systems) for the indicated time points.

For the *in vitro* proliferation assay, purified naïve CD4^+^ T cells were labeled with CellTrace™ Violet (Life Technologies) prior to stimulation and analyzed by flow cytometry 72 h post-stimulation.

### Homeostatic Proliferation

Naïve CD4^+^ T cells were purified from the spleens of WT and Asc^−/−^ mice, stained with CellTrace™ Violet and adoptively transferred into Rag1^−/−^ mice. After 4 days, spleens and mesenteric lymph nodes were harvested, and the single cell suspension was prepared. CD4^+^ T-cell proliferation was assessed by CellTrace™ Violet dilution and flow cytometry.

### T-Cell Transfer Colitis

Naïve CD4^+^ T cells (CD45RB^high^CD44^−^CD62L^high^CD25^−^) were sorted from splenocytes of WT and Asc^−/−^ mice using a BD Influx sorter and 3 × 10^5^ cells were adoptively transferred into Rag1^−/−^ mice (i.v). Some animals received a co-injection of 10^5^ sorted WT and Asc^−/−^ Tregs (CD4^+^CD25^high^). In this model, the co-transferred Tregs can suppress the expansion of activated CD4^+^ T cells in the gut hence preventing the onset of colitis. After 6 weeks, the mice were monitored weekly for weight loss. The mice were humanely euthanized 15 weeks post transfer by CO_2_ asphyxiation and macroscopic analysis, histopathological scoring and flow cytometric analyses of the leukocytes were performed. Stool consistency of fecal pellets was scored as follows: 0, normal stool; 1, soft stool but still formed; 2, very soft stool; 3, diarrhea; 4, liquid stool that sticks to the anus.

### Isolation of LP Cells From the Colon

The large intestine and cecum were harvested, and the fecal contents were removed by flushing with PBS. The colon was opened longitudinally, cut into 0.5–1 cm fragments and washed in RPMI medium supplemented with 2% FBS and incubated at RT for 20 min while stirring. The colon pieces were shaken vigorously three times for 30 s, followed by washing in calcium-free and magnesium-free PBS containing 1 mM EDTA for 40 min while stirring to remove the epithelium. The tissue pieces were then digested with 0.8 mg/ml collagenase type IV (Sigma-Aldrich) containing DNAase and 2% FBS at 37°C while stirring for 60 min. After filtering through a 70 μM cell strainer, the cells were spun down and enriched on a 40:75 percent Percoll gradient (Pharmacia). The interface ring containing the LP leukocytes was collected by centrifugation at 700 × *g* for 20 min.

### Histopathology

Colon sections were fixed in 10% formalin and cut into 5 μM thick sections before staining with hematoxylin and eosin. Stained sections were examined and scored in a blinded fashion. The degree of inflammation was graded semi-quantitatively from 0 to 4 as follows: 0, no signs of inflammation; 1, very low level; 2, low level of leukocyte infiltration; 3, high level of leukocyte infiltration, high vascular density, and thickening of the colon wall; and 4, transmural infiltration, loss of goblet cells, high vascular density, and thickening of the colon wall. The extent of inflammation was graded from 0 to 4 as follows: 0, none; 1, mucosal; 2, submucosal; 3, mucosal and submucosal; 4, full thickness. The overall inflammatory score was obtained by summing the “degree” and “extent” scores.

### *In vitro* CD4^+^ T-Cell Polarization

Sorted naïve CD4^+^ T cells (CD62L^high^CD44^−^CD25^−^) were cultured in the presence of 5 μg/ml plate-bound anti-CD3 (clone 2C11) and 2 μg/ml soluble anti-CD28 (clone 37.51) antibodies, and 20 ng/ml IL-2 (Th0). The following cytokine cocktails were used for T-cell polarization: Th1, 20 ng/ml IL-12 and 10 ng/ml anti-IL-4; Th2, 40 ng/ml IL-4, 10 μg/ml anti-IFNγ (clone R4-6A2), and 10 μg/ml anti-IL-12 (clone C17.8) antibodies; Th17, 2 ng/ml TGFβ, 100 ng/ml IL-6, 10 μg/ml anti-IL-4 (clone 11B11), 10 μg/ml anti-IFNγ (clone R4-6A2) antibodies, 20 ng/ml IL-23, 20 ng/ml IL-1β and 100 ng/ml IL-21; Th9, 10 ng/ml TGFβ, 10 ng/ml IL-4 and 10 μg/ml anti-IFNγ antibody (clone R4-6A2). The culture medium containing neutralizing antibodies only was refreshed on day 3. To assess T-cell polarization, the cells were re-stimulated on day 5 with 50 ng/ml PMA and 1 μg/ml ionomycin for 1.5 h followed by 5 μg/ml brefeldin A and 2 μM monensin for an additional 4 h. The cells were then fixed and permeabilized using a Foxp3 Staining Buffer Set (eBioscience) and stained with antibodies against IFNγ IL-4, IL-17, and IL-9 (all from eBioscience) and analyzed by flow cytometry.

For Treg-cell differentiation, naïve CD4^+^ T cells were cultured in the presence of 5 μg/ml plate-bound anti-CD3 (clone 2C11) and 2 μg/ml soluble anti-CD28 (clone 37.51) antibodies, 20 ng/ml IL-2, 5 ng/ml TGFβ, 10 μg/ml anti-IL-4 (clone 11B11) and 10 μg/ml anti-IFNγ (clone R4-6A2) antibodies for 3 days and then fixed and permeabilized using the Foxp3 Staining Buffer Set (eBioscience), and stained intracellularly with an anti-mouse Foxp3 antibody (clone FJK-16S, eBioscience).

### T-Cell Receptor Phosphor-Antibody Array Analysis

Sorted splenic naïve CD4^+^ T cells (CD44^−^CD62L^high^CD25^−^) from WT or Asc^−/−^ mice were stimulated with Mouse T-Activator CD3/28 Dynabeads^TM^ (ThermoFisher Scientific) at a bead to cell ratio of 1:1 for 30 min. The protein phosphorylation profiles of the unstimulated and activated T cells were assessed using a T-Cell Receptor Phospho Antibody Array Kit (Full Moon Biosystems).

### Flow Cytometry

For surface staining, cells were washed once with cold PBS and labeled with antibody cocktails for 30 min at 4°C. The cells were then washed with PBS and resuspended in FACS buffer (PBS containing 1% BSA, 1 mM EDTA, and 1 mM NaN3 Azide). DAPI was added to the samples 5 min prior to the acquisition. For intracellular staining, the cells were fixed and permeabilized using a FoxP3 Staining Buffer Set (eBioscience) and stained intracellularly, following the manufacturer's instructions. Prior to fixation, the cells were stained for 20 min at RT with Zombie UV Fixable Viability Kit (Biolegend) and labeled with the following fluorochrome-conjugated monoclonal anti-mouse antibodies: CD3, CD4, CD8, CD45, CD25, CD44, CD45RB, CD62L, CCR2, CD199 (CCR9), IFNγ, IL-17, IL-4, IL-9, Foxp3, Ly6C, Ly6G, MHCII, and CD11c (all from Biolegend). Cells were acquired on an LSRII flow cytometer (BD Biosciences).

For casp-1 and casp-8 activity assays, cells were labeled using the FAM-FLICA^TM^ Caspase-1 and Caspase-8 Assay Kits, respectively, for 1 h at 37°C, following the manufacturer's instructions (Immunochemistry).

### Western Blotting

Protein extracts were prepared using RIPA buffer and separated by 10–12% denaturing sodium dodecyl sulfate-polyacrylamide gel electrophoresis. Proteins were transferred onto nitrocellulose membranes, and membranes were incubated overnight at 4°C with the following primary antibodies: anti-Asc (1:1000, N-15, #sc-22514-R) and anti-Caspase-1 (1:1000, M-20, cat #sc-514) from Santa Cruz Biotechnology, and anti-GAPDH (1:2000, #MAB374, Millipore). The blots were developed using Western Lightning Plus-ECL Enhanced Chemiluminescence Substrate (Perkin Elmer).

### Enzyme-Linked Immunosorbent Assay (ELISA)

Cell-free supernatants from *in vitro* cultures or tissue homogenates were used to detect cytokines by ELISA or Luminex analysis using a 32 plex MILLIPLEX MAP Mouse Cytokine/Chemokine Magnetic Bead Panel (Millipore), following the manufacturer's instructions. IL-1β levels were assayed using a Mouse IL-1beta/IL1F2 DuoSet ELISA kit (R&D Systems). IL-18 levels were measured by Mouse IL-18 ELISA (MBL), as previously described (44). Caspase-1 levels were determined in the supernatants using a Caspase-1 ELISA Kit (AdipoGen).

### Metabolic Assays

Purified splenic naïve CD4^+^ T cells were stimulated with anti-CD3/CD28 antibodies for 16 h, and then plated on poly-Lysin-coated Seahorse XFe96 culture microplates (300,000 cells/well) in Seahorse XF Base Medium supplemented with glutamine (1 mM, for glycolysis stress test) or pyruvate (1 mM), glutamine (2 mM), and glucose (10 mM, for mitochondrial stress test). The cells were incubated at 37°C in an incubator without CO_2_ for 1 h prior to the assay. All assays were performed according to the manufacturer's instructions (Seahorse Bioscience); mitochondrial stress and glycolysis stress were measured as the oxygen consumption rate (OCR) (pmoles/min) and extracellular acidification rate (ECAR) (mpH/min), respectively, using an XFe96 extracellular flux analyzer (Seahorse Bioscience). Real-time injections for the glycolytic assay included glucose (10 mM), oligomycin (2 μM) and 2-deoxy-d-glucose (50 mM). For mitochondrial stress, the assay injections included oligomycin (1 μM), carbonyl cyanide-4-(trifluoromethoxy)phenylhydrazone (FCCP; 4 μM) and rotenone and antimycin (0.5 μM).

### Confocal Microscopy

Splenic naïve CD4^+^ T cells were incubated at 37°C in complete RPMI medium alone or with anti-CD3/CD28 antibodies for 72 h, with or without ATP (4 μM) for an additional 7 h. The cells were then fixed (4% PFA) for 30 min at room temperature, rinsed three times for 5 min and resuspended in PBS. For ASC and NLRP3 labeling, cytospin adherent cells were incubated in blocking buffer (0.1% saponin in 1% BSA-PBS) for 30 min at room temperature. The cells were then incubated with anti-mouse NLRP3 (Abcam #ab4207, Goat IgG 1:100) and/or anti-mouse ASC (#sc22514-R, Rabbit IgG, 1:30, Santa Cruz Biotechnology) diluted in blocking buffer for 3 h, followed by two washes in blocking buffer for 5 min and incubated overnight at 4°C. The next day the cells were washed once in blocking buffer for 5 min and incubated at room temperature for 1 h with the secondary antibodies diluted (1:500) in blocking buffer: mouse anti-rabbit IgG AF488 (Thermo Scientific) for ASC and donkey anti-goat IgG AF568 (Abcam) for NLRP3. The cells were then mounted with Prolong gold DAPI antifade reagent (Molecular Probes). For Ki67 staining, the cells were fixed, permeabilized, and labeled with Ki67 PE antibody (eBioscience, #SolA15).

### RNA Sequencing

Total RNA was extracted following the double extraction protocol by acid guanidinium thiocyanate-phenol-chloroform extraction (TRIzol, Thermo Fisher Scientific) followed by a Qiagen RNeasy Micro clean-up procedure (Qiagen). All mouse RNAs were analyzed on Agilent Bioanalyzer (Agilent) for quality assessment with RNA Integrity Number (RIN) range from 6.6 to 9.4 and median RIN 8.05. cDNA libraries were prepared using 1 ng of total RNA and 1 μl of a 1:100,000 dilution of ERCC RNA Spike in Controls (Ambion® Thermo Fisher Scientific) using SMARTSeq v2 protocol (45), except for the following modifications: 20 μM TSO and 250 pg of cDNA with 1/5 reaction of Illumina Nextera XT kit (Illumina). The length distribution of the cDNA libraries was monitored using DNA High Sensitivity Reagent Kit on the Perkin Elmer Labchip GX system (Perkin Elmer). All samples were subjected to an indexed PE sequencing run of 2 × 51 cycles on an Illumina HiSeq 2000 (21 samples/lane).

FASTQ files were mapped to the mouse transcriptome using Salmon based on GENCODE vM16 annotations. The counts were then used for differential gene expression analysis using the Bioconductor package edgeR running under R version 3.3.1. Gene Ontology enrichment analysis was conducted using topGO on the FDR corrected differentially expressed genes and heat maps generated using ComplexHeatmap (both topGO and ComplexHeatmap are Bioconductor packages). All data were available at the NCBI GEO database under the Accession Code GSE120928.

### Statistics

All data are expressed as the means ± SEM. An unpaired two-tailed student *t*-test was used for statistical analysis between two groups, and One-way ANOVA was used for comparisons of more than two groups. *P*-values ≤ 0.05 were considered statistically significant. GraphPad Prism version 6 (GraphPad Software) was used to prepare graphics and perform statistical analyses.

## Data Availability

The datasets generated for this study can be found in NCBI GEO database, Accession Code GSE120928.

## Ethics Statement

All experimental procedures were approved by the Institutional Animal Care and Use Committee (IACUC) of the Biological Resource Center (BRC), A^*^STAR in compliance with their Guidelines for Animal Experiments.

## Author Contributions

HJ and AMo: study concept and design. HJ, KL, AMe, MV, BM, KN, AT, FZ, and BL: acquisition, analysis, and interpretation of data. AMo: study supervision and fund acquisition. HJ and AMo: writing of the manuscript.

### Conflict of Interest Statement

The authors declare that the research was conducted in the absence of any commercial or financial relationships that could be construed as a potential conflict of interest.
