# Supplementary material for: The Inflammasome Adaptor ASC Intrinsically Limits CD4+ T-Cell Proliferation to Help Maintain Intestinal Homeostasis
Source: Front Immunol. 2019 Jul 15;10:1566. doi: 10.3389/fimmu.2019.01566 (PMC6644529; doi:10.3389/fimmu.2019.01566)
Supplement: Supplementary file 1 [file Data_Sheet_1.PDF]

SUPPLEMENTARY INFORMATION

The inflammasome adaptor ASC intrinsically limits CD4$^+$ T-cell proliferation to help maintain intestinal homeostasis

Hanif Javanmard Khameneh, Keith Weng Kit Leong, Andrea Mencarelli, Maurizio Vacca, Bezaleel Mambwe, Kurt Neo, Alicia Tay, Francesca Zolezzi, Bernett Lee, and Alessandra Mortellaro

SUPPLEMENTARY FIGURES

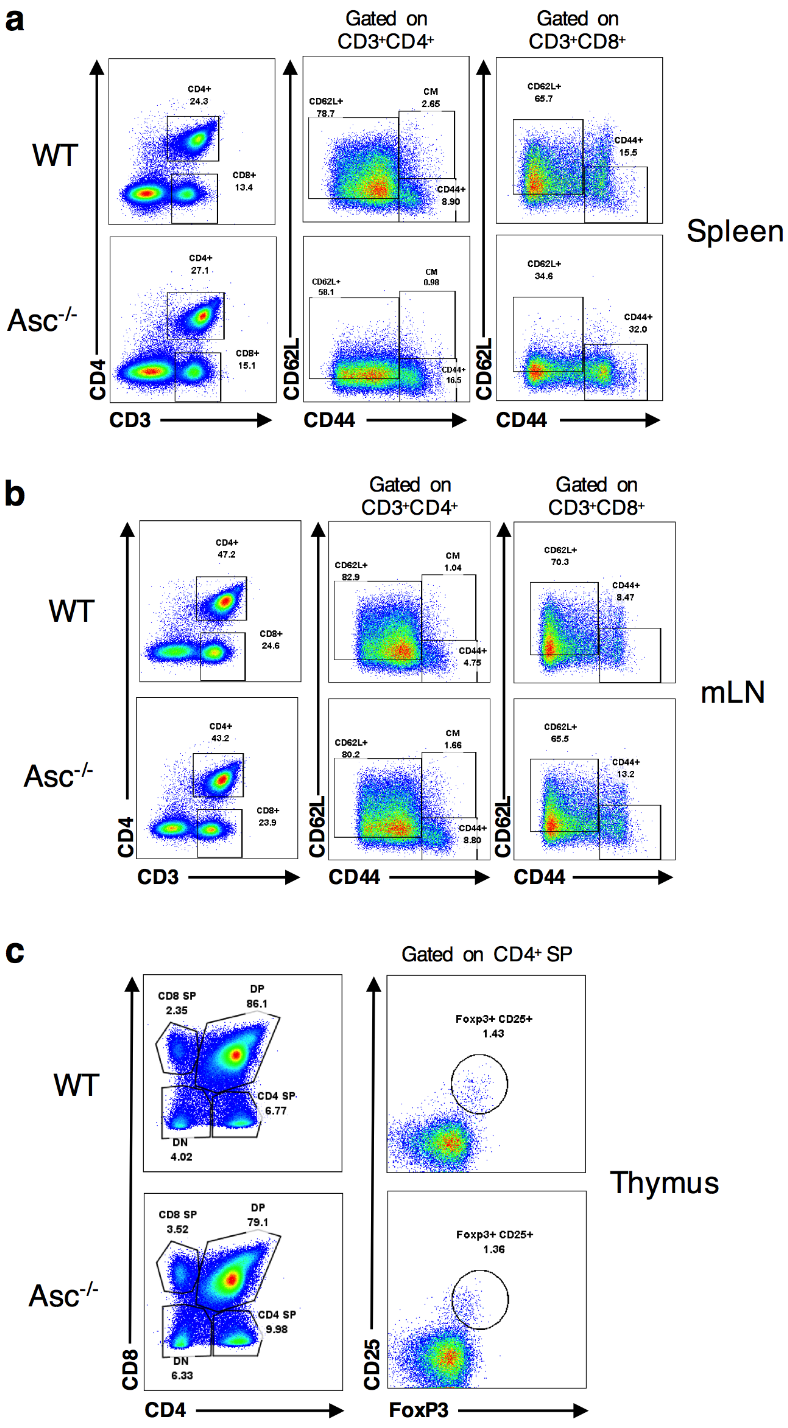

Supplementary Fig 1. CD4$^+$ T cells have an activated phenotype in the steady state in Asc$^{-/-}$ mice. Representative dot plots showing CD3$^+$/CD4$^+$, CD3$^+$/CD8$^+$ and naïve (CD44$^-$CD62L$^+$), effector (CD44$^+$CD62L$^-$), and central memory (CD62L$^+$CD44$^+$) T cells in (**a**) spleen and (**b**) mesenteric lymph nodes (mLN) of age-matched 8-10 weeks old WT and Asc$^{-/-}$ mice. (**c**) T cell and Treg (CD4^{sp}FoxP3$^+$) populations in thymi of WT and Asc$^{-/-}$ mice at 8-10 weeks of age.

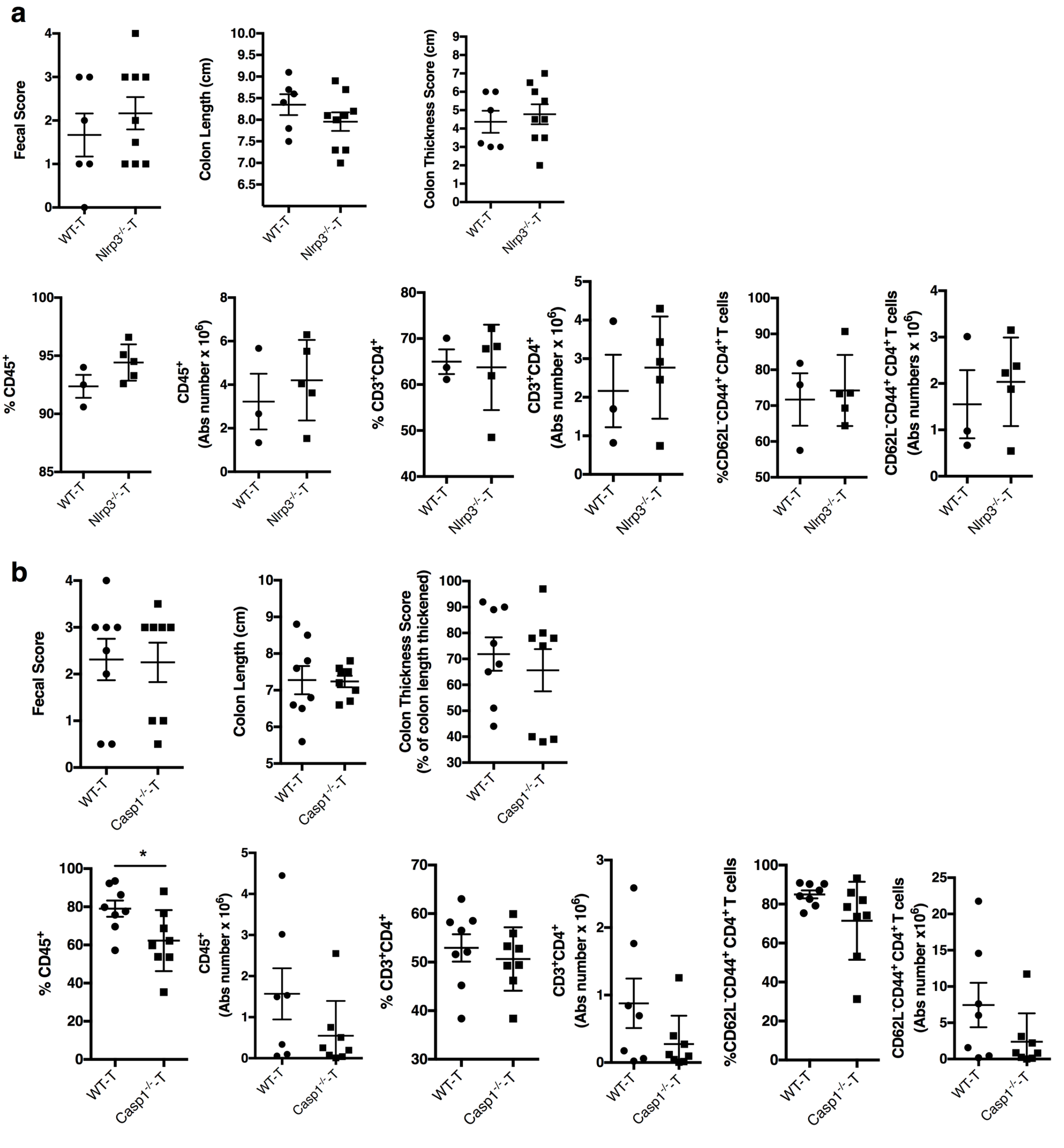

Supplementary Fig 2. Loss of NLRP3 and caspase-1 does not affect the colitogenic activity of $CD4^+$ T cells in vivo. Sorted splenic naïve $CD4^+$ T cells ($CD45RB^{high}CD62L^+CD44^-CD25^-$) ($3x10^5$/mouse) from wild-type (WT), $Nlrp3^{-/-}$ and $Casp\text{-}1^{-/-}$ mice were adoptively transferred into $Rag1^{-/-}$ mice. Recipient mice were sacrificed 13 weeks post-transfer, and the clinical scores and the cellular phenotypes of the colonic lamina propria (LP) cells were analyzed by flow cytometry. The stool consistency score, colon length, and thickness, percentages of total $CD45^+$ cells, $CD4^+$ T, activated $CD62L^-CD44^+CD4^+$ T cells and their corresponding numbers in colonic LP are shown. $Nlrp3^{-/-}$ (**a**) and $Casp\text{-}1^{-/-}$ (**b**) transfer colitis experiments are shown. The data represent the values of individual mice and the means ± standard error of a representative experiment (n = 9 mice).
